# Parotid Sialolithiasis in a 17-Year-Old Girl

**DOI:** 10.7759/cureus.36378

**Published:** 2023-03-19

**Authors:** Nawaf Almotairi, Mohammad Alotaibi, Manal Aldaihani, Mishal Almutairi

**Affiliations:** 1 Department of Otorhinolaryngology – Head and Neck Surgery, Farwaniya Hospital, Farwaniya, KWT

**Keywords:** minimally invasive surgery, sialendoscopy, sialolithiasis, salivary gland treatment, salivary glands

## Abstract

Sialolithiasis, caused by stones in the salivary glands or their excretory ducts, is one of the most prevalent salivary gland diseases. However, it is uncommon in the pediatric population and in the parotid gland. Both conservative and surgical methods are satisfactory options for sialolithiasis treatment. Small stones (<4 mm) located distal to the intraparenchymal gland often respond to conservative treatment. If medical treatment is anticipated to fail or the stones are medium or large sized (≥4 mm), surgery is the preferred option. This report describes a rare case of intraglandular parotid stones in an adolescent patient.

## Introduction

Sialolithiasis is a condition where the obstruction of the salivary gland or its excretory ducts occurs due to the formation of stones that lead to sialectasis, and the further dilatation of the gland may eventually cause chronic sialadenitis [1]. The main clinical symptoms are pain, fever, and swelling [2].

Sialolithiasis is more common in adults, with a higher prevalence in males than in females; however, the overall incidence is very low in the pediatric population [3]. The submandibular gland is the most common site of occurrence (80%-90%) of sialolithiasis, whereas the parotid gland is a less frequent site (10%-20%) [3]. Intraglandular parotid stones are less common than intraductal stones [4]. Parotid stones are usually smaller than submandibular stones, mostly <1 cm [1]. A thorough case history and clinical examination with imaging modalities should be performed to rule out various conditions when considering the differential diagnosis of sialolithiasis. Herein, we report a case of parotid sialolithiasis in a 17-year-old girl who presented with left parotid swelling. The diagnosis was confirmed using computed tomography (CT) of the neck, which showed intraglandular parotid stones, a rare occurrence among adolescent patients. This case report has been reported in line with the CARE (CAse REport) criteria [5].

## Case presentation

A 17-year-old, previously healthy, girl presented to the ear, nose, and throat (ENT) department at our hospital with a chief complaint of painful swelling in the left parotid region of the face for the past six months. The pain developed gradually, with a “pins and needles” sensation, and did not radiate to any other area. It was exacerbated by chewing food, especially sour foods, and alleviated by paracetamol. The pain was severe and peaked when the patient first woke up in the morning. Both pain and swelling subsided with the use of paracetamol. The pain usually subsided for approximately one hour after the intake of paracetamol, and then the pain resumed. These symptoms caused unintentional weight loss due to the inability to chew food. There was no history of trauma. Although the patient used anti-inflammatory medications, sialagogues, and antibiotics and maintained sufficient oral hydration, her symptoms persisted.

The patient’s previous medical, surgical, drug, and psychosocial histories were normal, and there were no known cases of the same illness in her family.

Clinical examination revealed swelling and tenderness in the left parotid gland. No discharge, skin changes, or temperature changes were observed over the swelling. The patient had good oral hygiene.

**Figure 1:**
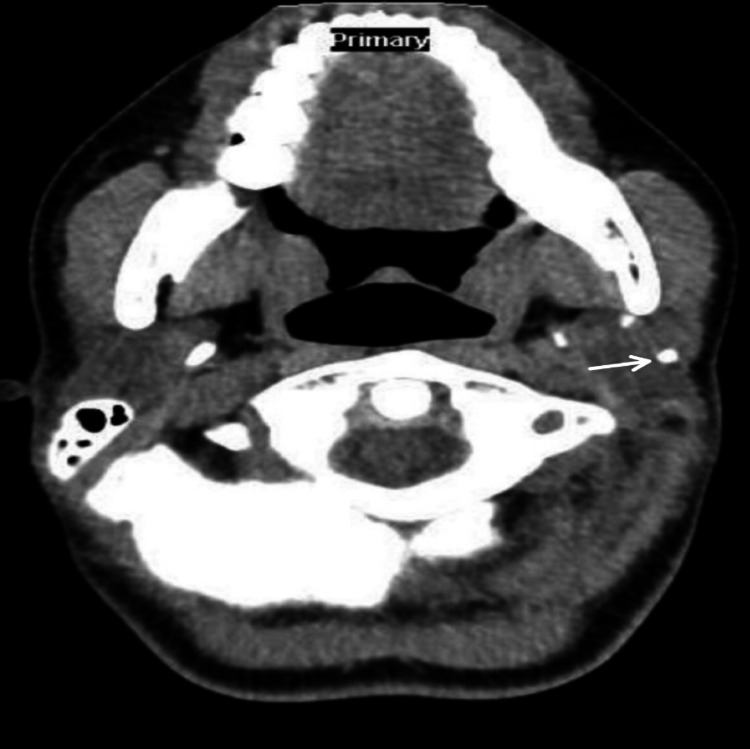
Axial computed tomography image showing the position of the salivary stone, measuring 3 mm, in the superficial lobe

**Figure 2:**
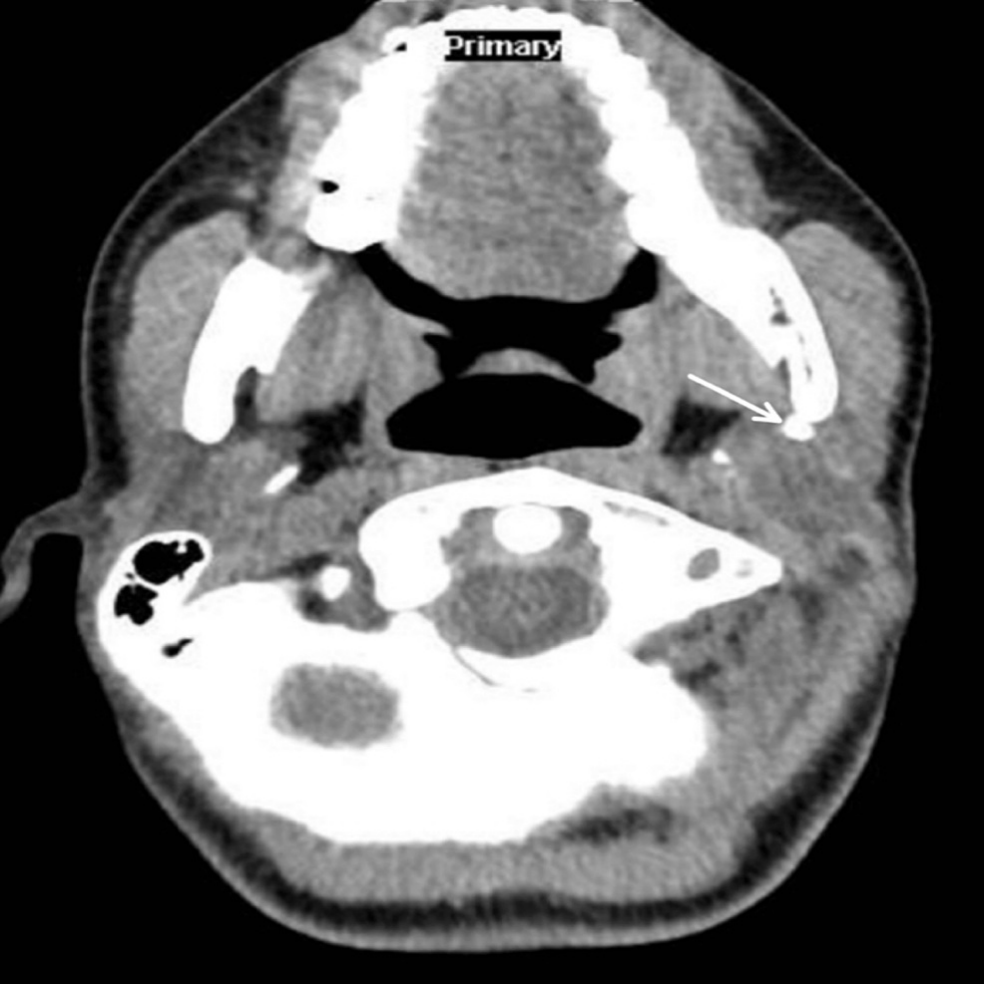
Axial computed tomography image showing the position of the salivary stone, measuring 5 mm, in the deep lobe

A CT of the neck showed evidence of two stones in the left parotid measuring 5 mm in the deep lobe and 3 mm in the superficial lobe. The left parotid gland was mildly swollen, with ductal dilatation (Figures 1, 2).

The patient’s laboratory results showed that the values for parathyroid hormone (11.7 pmol/L), 25-hydroxy vitamin D (20 nmol/L), and routine blood tests were within the normal range.

Management

The patient was operated on by a head and neck surgeon using a minimally invasive technique under general anesthesia with oral intubation. This extraoral procedure uses a facial nerve monitoring device and a small needle guided by a sterile ultrasound. With sterile ultrasound assistance, a small needle was placed outside the face until both stones were located and held in place. After performing a short pre-auricular incision for a parotid flap, to allow access to the parenchyma overlying the stone, the sub-superficial musculoaponeurotic system was dissected until the needles could be visualized using a zero-degree rigid scope. The stones were extracted after blunt dissection around the needle. The wound was closed using absorbable vicryl suturing 5.0, and a tight wrap was used to prevent saliva leakage. Anti-inflammatory medications were administered intravenously for four days. Following a four-day hospital stay, the patient was discharged symptom-free and had good overall health.

Follow-up

The patient recovered with complete remission of pain and swelling. Salivary glands functioned normally and with effective drainage. This was confirmed by postoperative ultrasonography of the neck. Three months after surgery, the patient was clinically well with no complaints of adverse effects.

## Discussion

Salivary stones are a rare occurrence in the pediatric population and in the parotid gland [6]. A literature search revealed that published reports of pediatric parotid gland sialolithiasis are limited to case reports. In our case, the patient was a 17-year-old girl; parotid gland sialolithiasis is uncommon at this age.

Although there is no apparent cause for sialolithiasis, Hathiramani et al. mentioned that some possible etiologies include altered salivary pH due to oropharyngeal infection, change in crystalloid solubility, blockage of salivary flow, and decreased salivary flow rate [7]. According to conventional beliefs, sialolith formation occurs in two stages. First, a central core composed of salts that bond to certain organic compounds during precipitation develops. Second, a periphery is formed via layer-by-layer deposition of organic and inorganic materials. Foreign objects, food, or bacteria that migrate backward from the oral cavity to the ducts of the salivary glands may serve as a nidus for producing stones via extra calcification [7].

The clinical signs and symptoms vary between patients with acute and chronic infections. In the acute phase, pain, swelling, and pus discharge are the predominant symptoms. In contrast, in the chronic phase, symptoms include recurrent swelling or non-resolving inflammation of the parotid gland [7].

Sialolithiasis has been diagnosed using both traditional and advanced imaging methods. Most submandibular and parotid stones are radiopaque, which may be observed on intraoral radiography [8]. Ultrasonography is a good first-line imaging modality because it can identify radiolucent stones > 1.55 mm in diameter with a 99% accuracy rate [9]. The masseter and buccinator muscles make it difficult to spot calculi in the parotid duct in the case of parotid stones. In these situations, sialography is a suitable technique that allows visualization of the entire duct and assessment of the stone size [7]. However, individuals with acutely infected salivary glands and those allergic to contrast agents cannot undergo sialography [3]. Notably, CT scans are superior to radiography and ultrasonography in diagnosing sialolithiasis and abscesses [10]. Although a CT scan may show abnormalities within the duct, it cannot pinpoint the exact location of the stone or reveal any structural ductal defect [11]. Magnetic resonance imaging (MRI) has many advantages over other imaging modalities, such as visualization of large stones, delineation of ductal anatomy, and assessment of the gland [7]. Due to its improved tissue discrimination, MRI can differentiate between acute and chronic blockages as well as identify glands with partial obstruction [12]. However, MRI takes longer and is more complicated and expensive than CT or radiography [13].

Conservative measures as well as surgical procedures are viable options for the management of sialolithiasis. Small stones (<4 mm) located distal to the intraparenchymal gland typically respond well to conservative therapy, which includes adequate hydration, massaging of the gland, sialagogues, anti-inflammatory drugs, and antibiotics [14]. Surgery is preferred if medical treatment is expected to fail or the size of the stones is medium or large (≥4 mm).

Surgical approaches are either intraoral or extraoral depending on the size, location (distal duct, hilar area, intraparenchymal ductal system), the positional relationship of the stones to the surrounding tissue (impacted, mobile, adhesive), and the number of stones [14]. The intraoral surgical approach is preferable for ductal stones, whereas the extraoral surgical procedure is helpful for intraparenchymal stones.

Extracorporeal shock-wave lithotripsy, sialendoscopy, and combined endoscopic and surgical procedures are alternative treatment options for sialolithiasis. However, they are not accessible to many institutions because of their high cost and the requirement for competent specialists. Additionally, their role in patients aged <18 years has not yet been studied [7]. Newer minimally invasive interventions have several advantages over invasive surgical methods due to their cosmetic benefits, shorter hospital stays, minimal visible scarring, and a lower risk of complications.

## Conclusions

Compared to submandibular stones, parotid stones are less common, especially in the pediatric population, are often unilateral, and more commonly involve the salivary duct than the gland. There are still no well-defined causes of sialolithiasis. Several diagnostic modalities are available to identify stones in the salivary glands. Sialolithiasis can be managed medically or surgically depending on the size and site of the sialoliths. Physicians should include sialolithiasis in their differential diagnosis of facial swelling of the parotid region regardless of the patient’s age.
